# Baptistæ Tinctoria

**Published:** 1847-12

**Authors:** 


*Baptistæ Tinctoria.*—DEAR DOCTOR: The following being scarcely worth a place as a separate article in your Journal, will answer as a note to your article on the *Sophora Tinctoria.*

Respectfully yours,
JAMES FOUNTAIN.

Taking a deep interest in the Medical Botany of my native country, I presume the following rough sketch will not be uninteresting to my fellow country physicians.

The plant commonly called Indigo Weed, the Baptista, or Sophora Tinctoria of Linn., grows so abundantly all over the country, is so easily recognised, and possesses such active properties, that it is not a little surprising that it has received so little attention.

About 30 years ago Dr. Thatcher noticed the plant in his Dispensatory, as being useful in cases of gangrene from deficiency of vital power, and in some other affections proceeding from the same cause.

In June, 1837, I was called to see Jacob Post, æt. 74, of an infirm habit. He had been a free liver, but was infirm from age, and was troubled with almost constant diarrhœa. He had a large purple patch on his leg, just above the outer ankle of his left leg. His foot was cold. He was not feverish, nor otherwise seemingly more indisposed than usual. He had no pain in his leg or foot, but only a slight soreness. In a few days his foot was spotted over with similar patches, and finally they all coalesced. The cuticle shortly pealed off, and the whole foot was covered over with a soft, black, grumous coating.

Warm stimulants and astringents were freely used externally, and tonics and opiates internally, but to no purpose; the gangrene spread gradually but slowly upwards, so that by the end of the month his leg as far up as the middle of the calf, had become one putrid mass, with no cognisable line of arrest, but gradually loosing its aspect upwards.

His leg had now become so intolerably offensive, that I

determined to remove it, to rid the family of the insupportable stench, for I had not entertained the least hope of saving his life.

On the 4th of July, my son amputated the leg below the knee. On removing the dressing, I found the surface of the stump, as I had anticipated, black enough. I now determined to try the indigo weed. I made a strong decoction of the fresh root, and with this I washed the part freely, and had the dressing wet continually. The gangrene soon spread up under the integuments, forming cavities and short sinuses. Into these I injected the decoction twice a day, freely.

The medicines administered internally were a decoction of oak bark with a few drops of laudanum, and a pretty free use of his favorite drink, brandy. Suffice it to say he finally recovered, and enjoyed excellent health and spirits something over three years, and finally died of influenza.

Another case of a Mr. Merrit, was on hand a few miles distant, almost precisely to this, in a subject about as old, but of a better constitution. Finding my case doing well his attendant amputated his leg, but it did not save him. He used only the means recommended in surgical works generally, but did not use the Tinctoria.

Since then a similar case on the back of the hand, and on one finger, came under the care of my son, of a very threatening aspect. It yielded to the Tinctoria similarly used.

Several minor cases have occured in my practice, tending to strengthen my confidence of the great value of the plant. Its activity has, as yet, prevented my using it internally. It doubtless possesses tonic, stimulant, and astringent properties in great activity, especially in its green state.

